# p62 Is a Potential Biomarker for Risk of Malignant Transformation of Oral Potentially Malignant Disorders (OPMDs)

**DOI:** 10.3390/cimb45090480

**Published:** 2023-09-19

**Authors:** Ryo Takasaki, Fumihiko Uchida, Shohei Takaoka, Ryota Ishii, Satoshi Fukuzawa, Eiji Warabi, Naomi Ishibashi-Kanno, Kenji Yamagata, Hiroki Bukawa, Toru Yanagawa

**Affiliations:** 1 Oral and Maxillofacial Surgery, Clinical Sciences, Graduate School of Comprehensive Human Sciences, University of Tsukuba, 1-1-1 Tennodai, Tsukuba 305-8875, Japan; 2 Department of Oral and Maxillofacial Surgery, Institute of Clinical Medicine, Faculty of Medicine, University of Tsukuba, 1-1-1 Tennodai, Tsukuba 305-8575, Japan; 3 Department of Biostatistics, Faculty of Medicine, University of Tsukuba, 1-1-1 Tennodai, Tsukuba 305-8575, Japan; 4 Department of Anatomy and Rmbryology, Institute of Medicine, University of Tsukuba, 1-1-1 Tennodai, Tsukuba 305-8575, Japan; 5 Department of Oral and Maxillofacial Surgery, Ibaraki Prefectural Central Hospital, 6528 Koibuchi, Kasama 309-1793, Japan

**Keywords:** p62, OPMDs, XPO1, ki67, p53, cancer development

## Abstract

To determine the intracellular behavior of p62, a marker of selective autophagy, in oral potentially malignant disorders (OPMDs). This retrospective study includes 70 patients who underwent biopsy or surgical resection and were definitively diagnosed with OPMDs. Immunohistochemical staining for p62, XPO1, p53, and ki67 was performed on all samples and positive cell occupancy was calculated. We statistically investigated the correlation between protein expression in OPMDs and the association between malignant transformation, clinicopathological characteristics, and occupancy. ki67 expression was negatively correlated with p62 expression in the nucleus (*p* < 0.01) and positively correlated with p62 expression in the cytoplasm (*p* < 0.01). For malignant transformation, the expression of p62 in the nucleus (*p* = 0.03) was significantly lower in malignant transformation cases, whereas the expression of p62 in the cytoplasm (*p* = 0.03) and the aggregation expression (*p* < 0.01) were significantly higher. Our results suggest that the function of p62 is altered by its subcellular localization. In addition, defects in selective autophagy occur in cases of malignant transformation, suggesting that p62 is a potential biomarker of the risk of malignant transformation of OPMDs.

## 1. Introduction

Oral potentially malignant disorders (OPMDs) are a newly defined concept by the World Health Organization (WHO), which defined them in 2017 as “clinical conditions that carry risk of cancer development in the oral cavity, regardless of whether they are clinically definite precursor lesion or normal mucosa” [1]. OPMDs include 12 diseases, including oral leukoplakia, oral lichen planus (OLP), and oral submucosal fibrosis. The malignant conversion rate of OPMDs is reported to be 7.9% [2]. One of the problems associated with OPMDs is their malignant nature; however, the actual causes of their pathogenesis and malignant transformation are not fully understood.

Anil et al. reported the presence and extent of epithelial dysplasia as predictors of the development of oral leukoplakia in cancer [3]. Although WHO has established a classification of epithelial dysplasia, its diagnosis is based on the subjectivity of the observer, thereby making uniform diagnosis difficult [4]. Therefore, to ensure objectivity in diagnosis, biomarkers must be developed to assess the risk of cancer development in OPMDs.

Treatment options for OPMDs include follow-up, removal of disease triggers such as inappropriate restorations and sharp edges of teeth, medication, and surgical resection [5]; however, there are no uniform treatment guidelines based on the subjective risk judgments of malignant transformation by medical professionals [6]. In addition, recurrence or malignant transformation may occur after surgical resection [7]. Identifying the biomarkers of developing cancer from OPMDs may lead to the development of treatments for OPMDs and may also lead to the prevention of oral cancer.

Selective autophagy is a mechanism that selectively degrades bacteria, specific organelles such as mitochondria, and aggregates of polyubiquitinated proteins [8]. Degradation by autophagy is usually nonselective; however, selectivity is achieved by specifically recognizing the degradation products [9]. Sequestosome-1 (p62) is a receptor protein that selectively incorporates ubiquitinated cargo into selective autophagy [10]. p62 contains a ubiquitin-associated domain (UBA) at its C-terminus, through which it can recognize and bind ubiquitinated proteins [11]; Additionally, p62 contains an LC3-interacting region (LIR). LC3 is an autophagy-associated protein that binds to isolated and autophagosome membranes [12]. This allows for ubiquitinated proteins to be selectively directed by p62 to the isolation membrane where they form autolysosomes and are degraded together with p62.

Recently, autophagy has been linked to various diseases, including Alzheimer’s disease, Parkinson’s disease, and cardiovascular disease [13]. It has also been reported that autophagy is impaired and in various malignant tumors, such as oral squamous cell carcinoma (OSCC), gastric carcinoma, and breast carcinoma, which results in the accumulation of p62 [14,15,16]. We previously reported that the expression of autophagy-related markers, including p62, at the resection margins of OSCC was associated with tumor recurrence [17]. We also found that p62 expression in the nucleus and p62 aggregation in oral leukoplakia were related to the presence or absence of epithelial dysplasia [18]. This suggests that p62, an adaptor of selective autophagy, may be useful as a marker for assessing the risk of recurrence at the resection margin of OSCC as well as the risk of malignant transformation in precancerous lesions.

Yoshida et al. reported the expression of p62 in oral leukoplakia; however, to the best of our knowledge, there are no studies on the expression of p62 in OPMDs [18]. Lin et al. reported that p62 showed a change in subcellular localization between oral mucosa and OSCC, suggesting that the subcellular localization of p62 changes in association with malignant transformation [19]. The purpose of this study was to reveal the intracellular behavior of p62 in OPMDs, with a focus on selective autophagy. We evaluated the intracellular expression of p62 in OPMDs and compared it with various clinical features, such as the malignant transformation of OPMDs, and biomarkers, such as Exportin-1 (XPO1), p53, and ki67, which are cancer-related factors.

## 2. Materials and Methods

### 2.1. Samples

Of the 104 patients who visited the Department of Oral and Maxillofacial Surgery at the University of Tsukuba Hospital between 2014 and 2021, underwent biopsy or surgical resection, and received a definitive diagnosis of OPMDs, 70 patients provided their informed consent to participate in the study. The specimens retrieved from the patients were formalin-fixed, and paraffin-embedded blocks were prepared for pathological examination. For patients who underwent both biopsy and surgical resection, the specimen obtained during surgical resection was used based on its condition. Clinical and clinicopathological data were obtained from medical records. All the specimens were diagnosed by at least two pathologists. Regarding malignant transformation of OPMDs, since OPMDs have the propensity to recur after surgical resection and is similar to OSCC, the following definition of local recurrence of OSCC was used as a reference: a local recurrence is defined as less than 2 cm away from or occurs within 3 years of the primary tumor and a second primary tumor as more than 2 cm from or occurs more than 3 years after the primary tumor in OSCC [20].

Clinicopathological characteristics including age, sex, drinking habits, smoking habits, location, disorder, presence of epithelial dysplasia, and development of cancer in the 70 cases included in the study are shown in Table 1. The median age of the patients included in the study was 64 years (range, 23–90 years) and the average age was 61.7 years at the time of treatment. Of the 70 patients, 50 had oral leukoplakia, and 20 had oral lichen planus. In addition, six cases of cancer developed after biopsy or surgical resection (8.6%).

This study was conducted in accordance with the Declaration of Helsinki and approved by the Institutional Review Board of the University of Tsukuba Hospital (approval no. R040-04).

### 2.2. Immunohistochemistry

All tissue sections had been fixed in formalin, paraffin-embedded, and sectioned to a thickness of 4 μm. Human liver cancer tissue sections were used as positive controls for p62, p53, and ki67, and human endometrioid adenocarcinoma tissue sections were used for XPO1 as specified in the protocol. Staining without primary antibodies was used as the negative control.

After deparaffinization and rinsing, samples were pretreated with a high pH system antigen activation solution (pH 9.0, K8004, Dako, Tokyo, Japan) in a microwave oven at 95 °C for 20 min. Endogenous peroxidase activity was removed by treatment with a mixture of 0.3% hydrogen peroxide in 100% methanol for 10 min. After thorough rinsing, the samples were incubated with primary antibodies against p62 (1:2000; PM045; MBL, Tokyo, Japan), XPO1 (1:250; D6V7N, #46249; Cell Signaling Technology, Tokyo, Japan), p53 (1:300; M7001; Dako), and ki67 (1:200; ab16667; Abcam, Cambridge, UK) for 60 min at room temperature, and ki67 (1:200; ab16667; Abcam) primary antibodies at room temperature for 60 min. The samples were washed and treated with a secondary antibody (Histofine Simple Stain MAX-PO(MULTI), Nichirei Bioscience, Tokyo, Japan) for 30 min at room temperature and treated with 3,3′-diaminobenzidine tetrahydrochloride (DAB, Cell Signaling Technology) for 5 min to detect antigen–antibody binding. Finally, hematoxylin was used to counterstain the nuclei.

### 2.3. Evaluation of p62, XPO1, p53, and ki67

**Figure 1:**
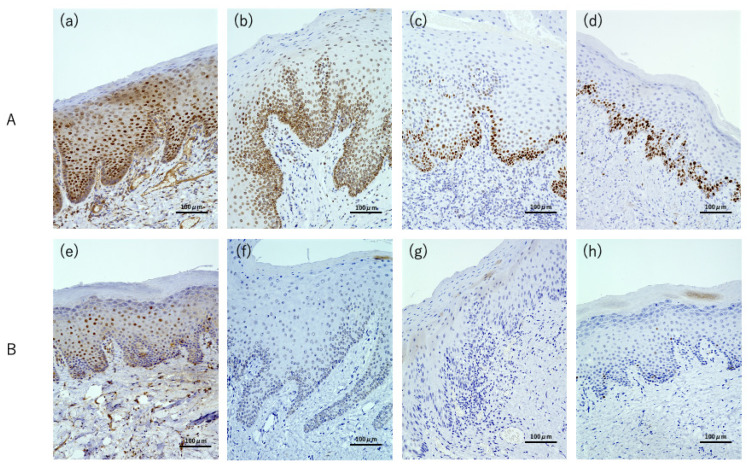
The result of p62, XPO1, p53, and ki67 immunohistochemical stains in OPMDs. (**A**) Representative sections showing strong expression of (**a**) p62, (**b**) XPO1, (**c**) p53, and (**d**) ki67. (**B**) Representative sections showing weak expression of (**e**) p62, (**f**) XPO1, (**g**) p53, and (**h**) ki67.

The expression of p62, XPO1, p53, and ki67 in the mucosal epithelium of the stained sections was evaluated. Although both strong and weak expressions were observed, we judged the cells as either positive or negative. A staining intensity equivalent to that of the positive control was considered positive. Images of representative strong and weakly expressing cases of each marker are shown in Figure 1.

All immunostained markers were assessed using an optical microscope (BZ-X710; KEYENCE, Osaka, Japan). Regions showing representative staining in sections were selected at a low-magnification field of view (5×) and evaluated at a medium-magnification field of view (20× and 40×). Three oral surgeons (RT, FU, and ST) determined the number of all epithelial cells and the number of positive cells for each marker at random. The positive cell occupancy rate for each was calculated, considering each receiver operating characteristic.

**Figure 2:**
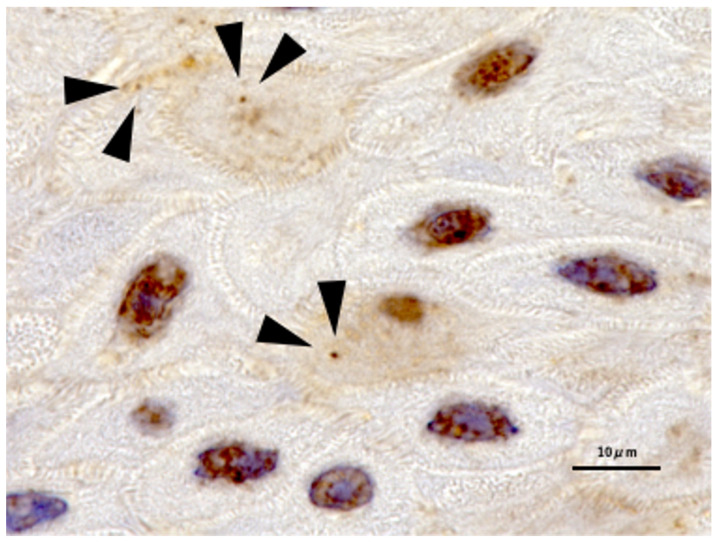
Representative example of p62 aggregation expression. p62 aggregation was defined as the presence of at least one dot indicating cytoplasmic accumulation. Arrows indicate representative p62 aggregation images.

Staining for p62 was evaluated separately for nucleus, cytoplasm, and aggregation. In accordance with our previous research, we defined a cell positive for p62 aggregation staining as one with at least one dot of accumulation in the cytoplasm in 20× and 40× field of view [18]. Representative images of p62 aggregation are shown in Figure 2. XPO1, p53, and ki67 were evaluated only for staining for nucleus.

### 2.4. Statistical Analysis

The correlation of protein expression in all OPMDs cases was evaluated using Spearman’s correlation test and is presented as a scatter plot. In addition, univariate analysis was performed using Fisher’s exact test with clinical characteristics and the Mann–Whitney U test for the association with each protein expression rate in cases of malignant transformation of OPMDs.

All *p* values less than 0.05 were considered statistically significant.

SPSS software ver. 28 (IBM Corp., Armonk, New York, NY, USA) was used for statistical analysis.

## 3. Results

### 3.1. Correlation of Each Protein Expression in OPMDs

**Figure 3:**
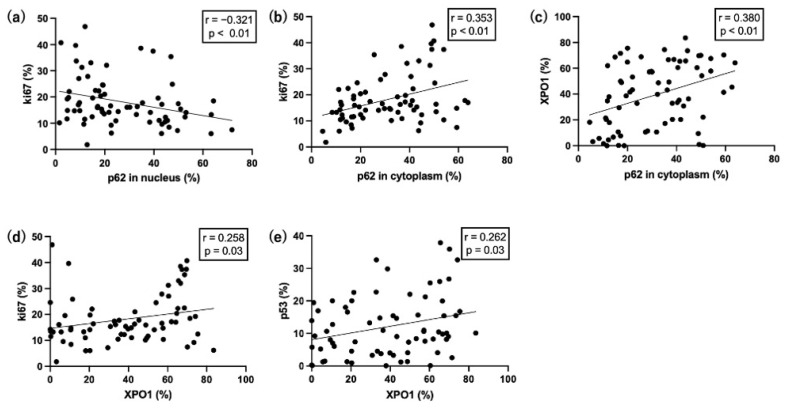
The correlation of each protein in OPMDs. In OPMDs, (**a**) p62 and ki67 were negatively correlated. (**b**) p62 in the cytoplasm and ki67, (**c**) p62 in the cytoplasm and XPO1; (**d**) XPO1 and ki67, and (**e**) XPO1 and p53 showed a positive correlation.

The expression levels of p62, XPO1, p53, and ki67 in OPMDs, which were correlated using the Spearman’s correlation test, are shown in Figure 3. The nuclear p62 expression was negatively correlated with ki67 expression (Figure 3a; r = −0.321; *p* < 0.01). The expression of p62 in the cytoplasm positively correlated with ki67 expression (Figure 3b; r = 0.353; *p* < 0.01) and XPO1 expression (Figure 3c; r = 0.380; *p* < 0.01). XPO1 expression positively correlated with ki67 expression (Figure 3d; r = 0.258; *p* = 0.03) and p53 expression (Figure 3e; r = 0.262; *p* = 0.03). The correlations for proteins other than these five were not significantly different.

### 3.2. The Association between Malignant Transformation of OPMDs and Clinical Characteristics or the Expression of Each Protein

The results of univariate analysis of the association between the malignant transformation of OPMDs and the clinical characteristics or the expression rate of each protein are shown in Table 2 and Table 3. No significant differences were found between malignant transformation and sex, age, drinking habits, smoking habits, or presence of epithelial dysplasia. Regarding protein expression, p62 in the nucleus (*p* = 0.03), p62 in cytoplasm (*p* = 0.03), and aggregation (*p* < 0.03) were associated with malignant transformation. Actually, the expression of p62 in the nucleus was significantly lower in the malignant transformation cases, whereas p62 expression in the cytoplasm and aggregation was significantly higher. In contrast, the expression levels of XPO1, p53, and ki67 were not associated with malignant transformation.

## 4. Discussion

This study yields two major findings. First, in OPMDs, nuclear p62 expression was negatively correlated with cell proliferation, whereas cytoplasmic expression was positively correlated with cell proliferation. In addition, p62 cytoplasmic expression correlated with XPO1 expression, suggesting that p62 may be transported to the cytoplasm by XPO1. In the cases of OPMDs malignant transformation cases, the p62 expression was altered in the subcellular localization from the nucleus to the cytoplasm, and the p62 aggregation expression was also significantly higher, suggested that selective autophagy is abnormal.

Selective autophagy plays a role in maintaining cellular homeostasis but is known to play a dual role in malignant tumors, depending on the type, stage, and genetic context of the cancer. In the early stages of tumorigenesis, it prevents chronic tissue damage by maintaining the genomic stability and by preventing the accumulation of carcinogenic mutations and inhibits the accumulation of cancer inducers. This enabled it to function as a cancer suppressor; however, in the late stages of tumorigenesis, it strengthens cancer cell survival and stress resistance by preventing tumor cell damage and satisfying high metabolic cravings. This eventually promotes tumorigenesis and leads to the accumulation of therapeutic resistance, which contributes to the progression and metastasis of malignant tumors [21]. In other words, at some stages of tumorigenesis, there is an alteration in the function and role of selective autophagy.

p62 is an adaptor protein for selective autophagy, which is resolved by autophagy along with its target proteins; therefore, the accumulation of p62 is thought to signify autophagy impairment [22]. Although intracellular protein metabolism involves the ubiquitin–proteasome and autophagy systems, when autophagy is impaired, p62 binds non-selectively to ubiquitinated proteins and prevents their transport to the proteasome, leading to the accumulation of ubiquitinated proteins and p62 [23]. Komatsu et al. reported that ubiquitinated proteins and p62 accumulate in autophagy-deficient mice and ubiquitin–p62 aggregates are markedly formed [24]. In addition, p62 aggregates promote intracellular accumulation by reducing the rate of nuclear-cytoplasmic shuttling owing to the large size of the polymer [25]. Therefore, the dynamics of p62 may reflect various intracellular signals, such as selective autophagy and nucleocytoplasmic transport signaling.

Originally, p62 was a shuttle protein that continuously and rapidly moved between the nucleus and cytoplasm, and this movement was regulated by the phosphorylation and aggregation of p62 [25]. Various studies have investigated the subcellular localization of p62; however, its function in the nucleus remains unclear.

ki67 is a protein expressed in all cell cycles except quiescence (G0 phase) and is used as a cell proliferation marker [26]. ki67 is seldom detected in normal cells but is highly expressed in most malignant tumors, including OSCC [27,28]. In this study, we found that in OPMDs, the nuclear expression of p62 was negatively correlated with cell proliferation, whereas the cytoplasmic expression of p62 was positively correlated with cell proliferation. Thus, there may be some connection between p62 in cytoplasm and cell proliferation. It has been reported that p62 accumulates in the cytoplasm rather than the nuclear in various types of malignant tumors [29,30]. Malignant tumors are characterized by abnormal cell proliferation, and the results of this study are consistent with those of previous studies. In addition, Iwadate, et al. reported cytoplasmic p62 expression may be involved in tumor growth and tolerance to cellular stress [31], which is also consistent with our study. These results suggest that changes in the subcellular localization of p62 may be associated with cell differentiation and proliferation.

XPO1 binds to the nuclear export signal (NES) of its target protein, forms a complex, and is transported from the nucleus to the cytoplasm via the nuclear membrane [32]. XPO1 participates in the transport of approximately 220 proteins via the NES [33]. XPO1 is overexpressed in many types of malignancies, and tumor proteins such as YAP1, c-ABL, and SNAIL, and tumor suppressors such as p53, p27, and RB are transported by XPO1 [32]. Therefore, XPO1 is a potential therapeutic target for malignant tumors. A multidomain protein containing various types of protein–protein interdomains, p62 has an NES and moves from the nucleus to the cytoplasm [25]. Previous report has also shown that p62 migrates and accumulates in the nucleus when gastric and liver cancer cells are treated with KPT-8602, an XOP1 inhibitor [34]. Herein, the cytoplasmic expression of p62 correlated with XPO1 expression, indicating that p62 may be transported to the cytoplasm in association with XPO1. Therefore, it was predicted that XPO1 overexpression in malignant tumors causes p62 to move from the nucleus to the cytoplasm, resulting in a change in its subcellular localization.

**Figure 4:**
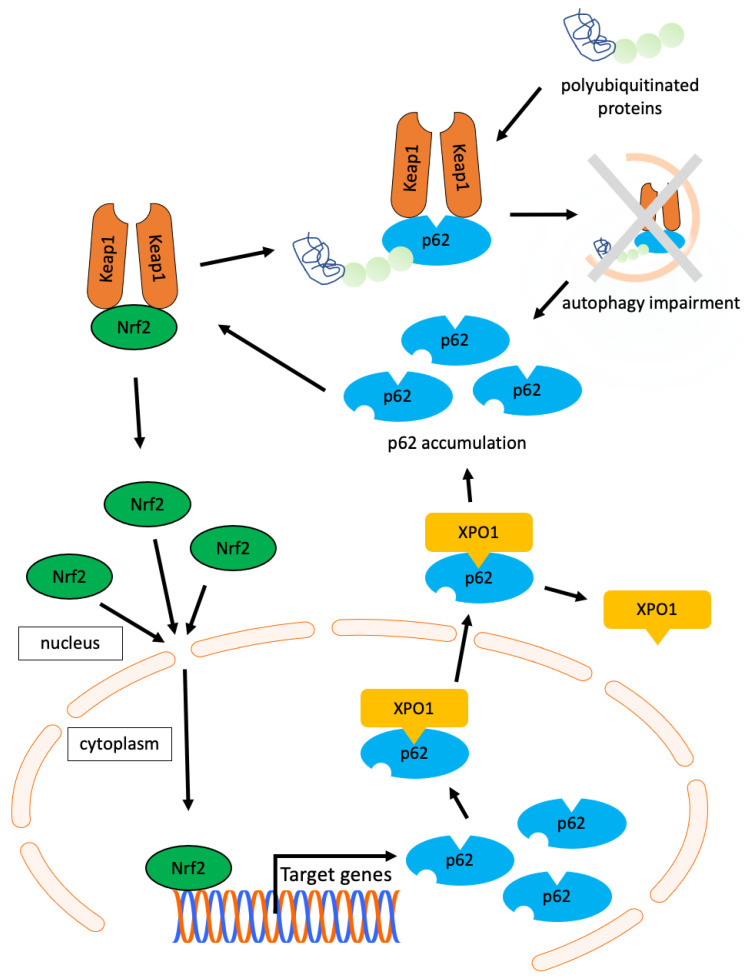
Diagram of the relationship between p62, XPO1, and autophagy in OPMDs as predicted by the results herein. The Keap1–Nrf2 complex separates in the presence of p62, Keap1 binds tightly to p62, and the activated Nrf2 translocate to the nucleus. In the nucleus, Nrf2 induces the expression of several target genes, including p62. Nuclear p62 binds XPO1 and translocate to the cytoplasm. Normally, polyubiquitinated proteins are digested together with p62 by selective autophagy, and p62 is thought to accumulate due to impaired autophagy.

In addition to autophagy, p62 is involved in the Keap1-Nrf2 pathway [11]. p62 has a Keap1-interaction region that binds to the Keap1 domain involved in its interaction with Nrf2. Thus, p62 overexpression inhibit the binding between Keap1 and Nrf2 and activated Nrf2. Activated Nrf2 translocates to the nucleus and induces the expression of several Nrf2 target genes, including p62. This activates a positive feedback mechanism for p62, and p62 accumulation progresses [35]. In addition, p62 is not metabolized in the presence of autophagy disorders and further accumulation occurs. A schematic representation of the relationship between p62, XPO1, and autophagy in OPMDs and a positive feedback mechanism of p62 is shown in Figure 4.

p53 acts as a tumor suppressor by inducing genes involved in cell cycle arrest, apoptosis, senescence, and repairing DNA [36]. p53 is the most frequently mutated protein in OSCC [37]. In addition, mutant p53 in patients are associated with decreased survival and resistance to radiotherapy and chemotherapy. Normally, p53 is degraded so rapidly that it cannot be detected by immunohistochemistry (IHC); however, mutant p53 has an extended half-life and can be detected by IHC [38]. XPO1 and ki67 are correlated with colorectal cancer. This suggests that the overexpression of XPO1 leads to uncontrolled cell division and tumor growth through subcellular localization changes in cell cycle inhibitory proteins, such as p21 and p53, and apoptotic proteins [39]. In this study, the correlations between XPO1 and p53 and between XPO1 and ki67 were shown in OPMDs, which correspond to this report.

Several studies have reported changes in p62 subcellular localization in normal tissues and malignant tumors. Lin et al. reported no difference in p62 nuclear expression but significantly increased the p62 cytoplasmic expression in OSCC compared to normal oral mucosa. The combination of the high p62 cytoplasmic expression and the low p62 nuclear expression in OSCC is also associated with a lower overall survival and disease-specific survival [19]. Kitamura et al. found that the p62 nuclear expression decreased and the cytoplasmic expression increased in malignant prostate tissues compared to benign prostatic tissues, suggesting that p62 migrates from the nucleus to the cytoplasm following tumorigenic transformation [30]. Interestingly, in cases of cancer development in OPMDs, p62 showed a change in subcellular localization from the nucleus to the cytoplasm compared with non-cancer development cases, which is similar to the results of these studies. In addition, no significant difference in the XPO1 expression was observed in cases of malignant transformation of OPMDs, suggesting that the nucleocytoplasmic transport system was not abnormal. p62 aggregation was also significantly increased, suggesting impaired autophagy. In other words, these results suggest that in cases of cancer development in OPMDs, some abnormalities in autophagy, increased cytoplasmic expression of p62, and decreased nuclear expression of p62 occur prior to the actual malignant transformation.

This study has several limitations. First, in this study, 6 cases (8.6%) were malignantly converted, which is similar to the malignant conversion rate reported previously [2]. However, this study was conducted at a single institution, which may have limited the sample size and selection of cases, leading to bias. Second, there are no uniform standards for immunostaining. To implement these biomarkers, further studies should be conducted using uniform criteria to obtain quantitative data. Third, regarding the correlation of expression proteins in OPMDs, the correlation coefficients ranged between 0.25 and 0.38, suggesting that the correlation is weak. However, in reality, various factors are expected to be involved in protein expression, and the correlation may have decreased possibility; therefore, a complex clarification of protein expression is a future issue. Finally, further studies are needed to clarify the diverse functions of p62, as this study focused on autophagy. For example, as mentioned in Figure 4, the intercellular behavior of p62 also involves the Nrf2-Keap1 pathway, which is an interesting topic. However, this is the first study to examine the association between the expression of p62, an autophagy-related protein, and cancer development in OPMDs. In particular, the subcellular localization of p62 may serve as a biomarker for assessing the risk of cancer development in OPMDs. Further investigation is needed to elucidate the mechanism of the malignant transformation of OPMDs and identify new biomarkers.

## 5. Conclusions

In this study, we found that the properties of p62 in OPMDs with respect to cell proliferative capacity were reversed between the nuclear and cytoplasmic expression. In addition, in cases of malignant transformation of OPMDs, the subcellular localization of p62 changes from nuclear to cytoplasmic, and p62 aggregation increases, suggesting that selective autophagy is impaired. This indicates that changes in the subcellular localization of p62 may be a potential biomarker for assessing the risk of malignant transformation in OPMDs.

## Figures and Tables

**Table 1:** Clinical characteristics of 70 cases.

Characteristics		Cases (%)
Sex	Male	47 (67.1)
	Female	23 (32.9)
Age	65>	37 (52.9)
	65≤	33 (47.1)
Drinking	Yes	51 (72.9)
	No	19 (27.1)
Smoking	Yes	43 (61.4)
	No	27 (38.6)
Location	Tongue	24 (34.3)
	Others	46 (65.7)
Disorder	Leukoplakia	50 (71.4)
	OLP	20 (28.6)
Epithelial dysplasia	Positive	53 (75.7)
	Negative	17 (24.3)
Developed Cancer	Yes	6 (8.6)
	No	64 (91.4)

**Table 2:** Relationships between cancer development of OPMDs and clinical characteristics.

Characteristics		Developed Cancer Cases (%)	No Cancer Cases (%)	*p*
Sex	Male	3 (6.4)	44 (93.6)	0.39
	Female	3 (13.0)	20 (87.0)	
Age	65>	1 (2.7)	36 (97.3)	0.09
	65≤	5 (15.2)	28 (84.8)	
Drinking	Yes	4 (7.8)	47 (92.2)	0.66
	No	2 (10.5)	17 (89.5)	
Smoking	Yes	3 (7.0)	40 (93.0)	0.67
	No	3 (11.1)	24 (88.9)	
Location	Tongue	3 (12.5)	21 (87.5)	0.41
	Others	3 (7.0)	43 (91.5)	
Disorders	Leukoplakia	4 (8.0)	46 (92.0)	1.00
	OLP	2 (10.0)	18 (90.0)	
Epithelial dysplasia	Positive	2 (13.3)	13 (86.7)	0.60
	Negative	4 (7.3)	51 (92.7)	

**Table 3:** Relationship between cancer development of OPMDs and the expressions of each protein.

Parameter	Developed Cancer Expression (%)	No Cancer Expression (%)	*p*
p62 in nucleus	12.9 ± 5.9	29.7 ± 19.0	0.03 *
p62 in cytoplasm	45.4 ± 12.5	29.8 ± 15.5	0.03 *
p62 aggregation	6.5 ± 3.6	2.1 ± 1.9	<0.01 *
XPO1	41.6 ± 22.9	38.9 ± 25.0	0.78
p53	14.2 ± 8.5	11.9 ± 9.4	0.44
Ki67	20.3 ± 6.5	18.0 ± 9.7	0.20

* *p* < 0.05 significant.

## Data Availability

The data presented in this study are available upon request from the corresponding author. The data are not publicly available because of the lack of conflicts of interest.
